# Microdialysis in the femoral head of the minipig and in a blood cloth of human blood

**DOI:** 10.3109/17453674.2011.566132

**Published:** 2011-04-05

**Authors:** Morten Foged Bøgehøj, Claus Emmeluth, Søren Overgaard

**Affiliations:** Odense University Hospital, Odense, Denmark

## Abstract

**Introduction:**

Microdialysis can detect ischemia in soft tissue. In a previous study, we have shown the development of ischemia in the femoral head removed from patients undergoing total hip replacement. That study also raised some methodological questions that this study tries to answer: what is happening in the dead space around the catheter in the drill canal, and is there an equilibrium period after the insertion of the catheter?

**Material and methods:**

In an ex-vivo study using 5 syringes with 5 mL human blood, a microdialysis catheter was inserted and microdialysis was performed over 3 h. In an in-vivo study, a drill hole was made in the proximal part of the femur in 6 mature Göttingen minipigs and microdialysis was performed over 3 h. The pigs were kept normoventilated during the experiment.

**Results:**

The ex-vivo microdialysis results showed that lactate kept a steady level and glucose and glycerol both fell; pyruvate fell but leveled out. The mean lactate/pyruvate ratio increased from 13 (SD 4) to 32 (SD 6) (p < 0.001). In vivo, relative recovery was 57% (SD 11). Lactate increased, pyruvate stayed constant, and glucose and glycerol levels fell. The lactate/pyruvate ratio increased from 30 (8) initially to 37 (8) after 1 h (p = 0.007) but no statistically significant change from 1 to 2 h was observed.

**Interpretation:**

The ex-vivo study showed a clear washout pattern, and was different from what we see in bone. The in-vivo study indicated that an equilibrium period is necessary or that a reference measurement in healthy bone must be used when performing short measurements in bone.

Microdialysis enables measurement of the concentrations of molecules in the extracellular fluid by the use of a catheter with a semipermeable membrane. This gives a unique opportunity to monitor local changes in metabolism (Rosdahl et al. 1993). The technique has been widely used to monitor metabolism and ischemia in soft tissue—for example, the brain (Skjoth-Rasmussen et al. 2004), skeletal muscle (Muller et al. 1996, Korth et al. 2000), and adipose tissue (Rosdahl et al. 1993).

The use of microdialysis in bone has been limited, and only measurements of prostaglandins (Thorsen et al. 1996) and gentamycin (Stolle et al. 2003, 2004) have been reported. We have established the method in human bone and have shown ischemia in human femoral heads removed during total hip replacement (Bøgehøj et al. 2007).

Bone viability might also be detectable by measuring the blood flow with a laser Doppler probe, which has been used for measuring blood flow in human bone. It has shown valid results, with good reproducibility and results that are in accordance with other methods for measuring perfusion in bone, e.g. microspheres (Swiontkowski et al. 1986, El Maraghy et al. 1999, Notzli et al. 2002, Beck et al. 2004).

Microdialysis in bone raises some methodological problems, namely dead space around the catheter and the effect of drilling on the local microenvironment. Thus, in this study we aimed (1) to study the development of ischemia and washout of metabolites in a blood clot (dead space) as a control for the in-vivo experiments, and (2) to perform in-situ microdialysis in the femoral head of a minipig to monitor the development in concentrations of the metabolites, in order to investigate the equilibrium period.

## Material and methods

### Ex vivo, the blood clot

The study was carried out on 5 different samples. 5 mL of human blood was collected in a 5-mL syringe. A CMA 70 catheter (20 mm membrane length, 20 kD cut-off; CMA, Solna, Sweden) was inserted through the opening of the syringe, and the catheter was connected to a CMA 107 pump with the flow rate set to 2 μL/min. The catheter was flushed for 3 min. Then samples were collected over 5 min at the times of 00:05, 00:20, 00:40 etc. for 3 h. The syringes were left at room temperature (23°C) during the sampling period and the samples were analyzed on a CMA 600 microdialysis analyser immediately after the experiment was ended.

### Drill holes in vivo

6 female Göttingen minipigs (Ellegaard Göttingen Minipigs ApS, Denmark) were included. All pigs were skeletally mature with a median age of 39 (25–49) months and a median weight of 46 (40–51) kg.

### Anesthesia

As premedication, 0.06 mg/kg medetomidine, 0.2 mg/kg midazolam, and 0.03 mg/kg atropin were given intramuscularly. Induction of anesthesia was done with 2 mg/kg propofol given intravenously. The pigs were intubated and ventilated with a frequency of 10 breaths/min and a tidal volume of 4–5 mL/kg with 2% sevofluran in a 1:1 mixture of O_2_: N_2_O. A Dameca ventilator was used (Dameca, Denmark). After surgical preparation, infusion of ketamin (7 mg/kg/h) was started and sevofluran was reduced to 1%. After the experiments, the animals were killed using an injection of 2 g phenobarbital. Isotonic NaCl infusion was given during the surgery.

During surgery and sampling, the minipigs were monitored with invasive blood pressure measurements, ECG, oxygen saturation, and central temperature (Infinity Gamma; Dräger Medical, Germany). Blood gasses were collected and analyzed every 30 min using a Radiometer blood gas analyzer (Radiometer Copenhagen, Denmark). PCO_2_, PO_2_, and pH were monitored in order to achieve steady state and normal ventilation. Only adjustment of the tidal volume from 200 to 250 mL/breath was necessary during the anesthesia to keep the minipigs normoventilated (defined as pH = 7.43 ± 0.03 and pCO_2_ = 40 ± 3 mmHg) (Svendsen and Carter 1989). In addition, arterial plasma glucose concentration was monitored every 30 min. The pigs all had a central temperature of close to 35°C during the sampling period.

### Surgery

The minipig was positioned in lateral decubitus. Using a lateral approach, the right proximal femur was exposed. A cleavage in vastus lateralis was made digitally and a drill hole of 2 mm in diameter and a depth of 30 mm was made into the center of the femoral neck under fluoroscopic control. Then laser Doppler measurements and microdialysis were performed.

### Laser Doppler

A laser Doppler probe (a DP3 probe connected to a DRT4 laser Doppler v. 5.02 (Moor Instruments, Axminster, UK) was introduced in order to demonstrate pulsatile flow. Data were collected using moorSOFT DRT4 monitor for Windows version 2.0 (Moor Instruments 2003).

We defined pulsatile flow when the following requirements were fulfilled:

1. A steady DC curve. This gives an indication of the backscattered laser light intensity. It can be used to check the efficiency of light collection by the laser Doppler probes.
2. A pattern of waves with a uniform appearance.
3. Pulse synchronous waves.
4. Mean flux > 10 AU (Gamse and Saria 1987).
5. ΔFlux of minimum 8 AU (amplitude).

Mean flux (SD), maximum flux, and minimum flux were measured over a period of 10 seconds using the statistics function in the DRT4 monitor software. From this, the ΔFlux was calculated (Bøgehøj et al. 2007).

### Microdialysis

A microdialysis catheter (CMA 70) was inserted. The catheter was connected to a CMA 107 microdialysis pump. The flow rate was 2 μL/min. The catheter was flushed for 3 min to empty the efferent tube. Then samples were collected over 5 min at times of 00:05, 00:10, 00:20, 00:40, 01:00, 01:20, etc. for 3 h. By the end of the experiment, the position of the catheter was checked visually and then removed and inspected; all catheters were found to be in place with no visible damage. The samples were stored at room temperature and analyzed on a CMA 600 microdialysis analyzer immediately after surgery.

The relative recovery (RR) was calculated using the mean of the 2 first microdialysis samples, collected from time 00:00 to 00:05 and from 00:05 to 00:10, and the arterial blood sample collected during the same period. RR was calculated using the glucose concentration of the arterial blood sample divided by the mean concentration of the first 2 samples from microdialysis.

### Statistics

Data are presented as mean (SD). To evaluate the changes in the concentrations of substances, a comparison of initial and stable-level substance concentrations was done using a paired t-test. Stable was defined as the last time point after which the measurement level decreased. Initial measurements were taken as the mean of samples 1 and 2. The level of significance was taken to be 0.05. The tests were performed using StataT SE version 9.2.

## Results

### Blood clot study

The results from this part of the study had 3 outliers, which were due to technical errors. They were excluded; they all came from the same microvial.

**Figure 1.**
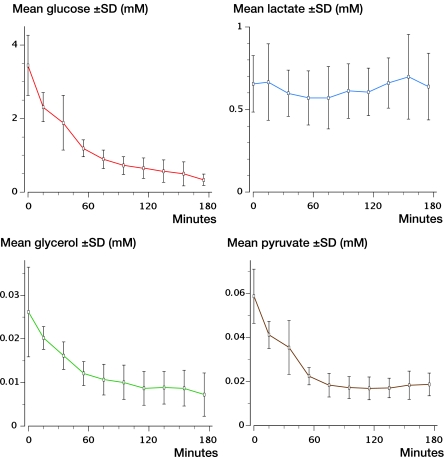
Results from blood clot study (n = 5): mean glucose, lactate, glycerol, and pyruvate levels. (Error bars show SD).

**Figure 2.**
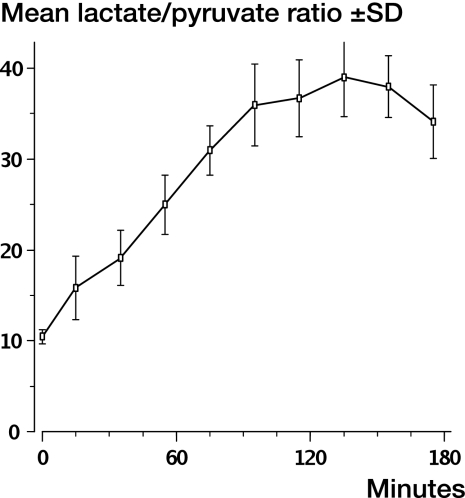
Results from blood clot study (n = 5): mean lactate/pyruvate ratio. (Error bars show SD).

Lactate showed a steady level of around 0.62 (0.05) mM (p = 0.08). Pyruvate started at 0.049 (0.013) mM and fell over the first hour, to around 0.019 (0.005) mM (p < 0.001), which was followed by a stable period (Figure 1). Lactate/pyruvate ratio increased from 13 (4) to 32 (6) (p < 0.001) (Figure 2).

The values for both glycerol and glucose fell significantly with time (Figure 1).

### Drill holes

The first minipig was hyperventilated, but during the start of the anesthesia the ventilator was adjusted according to the blood gases, and normal values were obtained and maintained for the rest of the experiment. Microdialysis was started when the minipig was normoventilated. The other 5 minipigs were kept normoventilated from the start to the end of the experiment.

All drill holes showed pulsatile flow according to our definition, with a mean flux of 35 (7) AU.

The mean plasma glucose in arterial blood increased over time from 4.3 (2.0) mM to 5.8 (1.6) mM.

**Figure 3.**
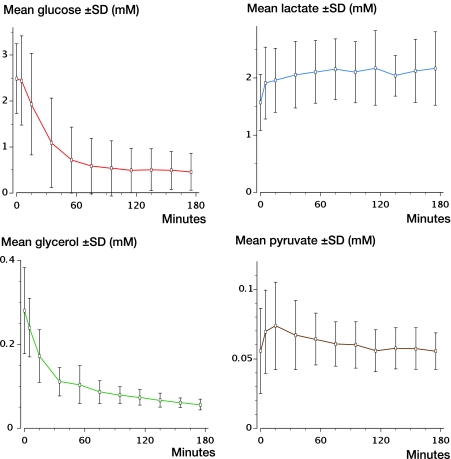
Results of microdialysis in a drill hole in the femoral heads of minipigs (n = 6): mean glucose, lactate, glycerol, and pyruvate levels. ((Error bars show SD).

Microdialysis showed that lactate increased from 1.7 (0.6) mM to 2.2 (0.6) mM whereas pyruvate values started at 0.063 (0.03) mM and fell to 0.056 (0.013) mM after a small rise at 20 min, of 0.074 (0.031) mM (Figure 3).

**Figure 4.**
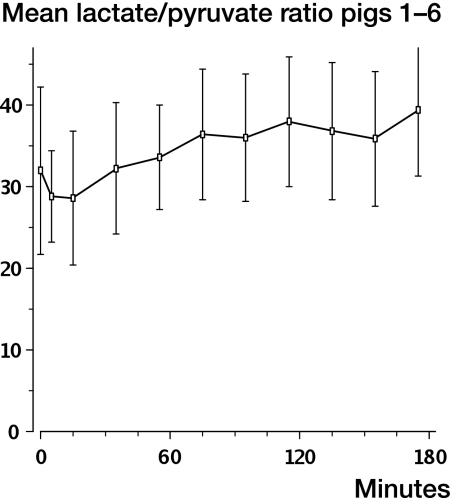
Mean lactate/pyruvate ratio for pigs 1–6. (Error bars show SD).

Lactate/pyruvate ratio reached a steady state after 140 min. The difference from initial values followed a Gaussian distribution, as indicated in a probit plot. The mean difference was 6.4 (95% CI: 1.8–11) (p = 0.02, one-sample t-test).

The values for glucose and glycerol fell rapidly over the first hour and then leveled out, with a slight falling trend. Glucose started at 2.5 (0.8) mM and fell to 0.6 (0.6) mM (p < 0.001) over the first 2 h whereas glycerol fell from 0.26 (0.09) to 0.09 (0.03) (Figure 3). Relative recovery (RR) was 57% (11).

## Discussion

This study was done to evaluate and validate findings from microdialysis in bone of patients undergoing total hip replacement (Bøgehøj et al. 2007). In order to do microdialysis in bone, a drill hole has to be made—which imposes trauma on the surrounding bone influencing the local microenvironment. The drill hole has to be bigger than the catheter in order to insert the catheter without damaging it. This creates a dead space around the catheter, in which the only transport of substances is diffusion.

The ex-vivo part of our study was done on a blood clot in order to show whether there was washout, and to compare the difference between measurements made in the dead space around a catheter and measurements made in bone. In the blood clot, the only substrate to increase was lactate due to the anaerobic conditions where the metabolism generates lactate. In contrast, pyruvate, glucose, and glycerol levels fell over the study period. This can be explained by washout, where the microdialysis catheter removes more of the metabolites than can be produced or delivered to the tissue. Thus, equilibrium cannot be maintained and we found an artificially low concentration of these substances. In the ex-vivo study, no more substances are delivered and the washout pattern is expected. The lactate concentration was kept at a steady level, which is explained by a continuous supply due to anaerobic conditions in the syringe.

In order to be able to rely on the data sampled during the study, the pigs had to be as close to normoventilated as possible. If the pH value drifts or the pCO_2_ falls to very low levels, the conditions will not be as in the routine clinic. This is why we strictly controlled the blood gas samples and adjusted the ventilator accordingly.

The in-vivo study showed that the pyruvate concentration stayed almost constant with a slight tendency to decrease, although this was not statistically significant during the study, whereas lactate showed a small increase. This indicates that the measurements obtained in the bone reflect the metabolism within the bone and not a washout pattern as seen in the blood cloth. This suggests that an equilibration period of about 2 h is needed.

The lactate/pyruvate ratio was 2-fold higher compared to human data (Bøgehøj et al. 2007), but this has been seen in other minipig studies (unpublished data)—which might be explained by the fact that minipigs have a higher level of plasma lactate. We consider that it is not the actual level but any development in the ratio that is important. A ratio above 25 is considered ischemic in human tissue (Ungerstedt and Rostami 2004).

Glucose and glycerol concentrations fell statistically significantly, which might be explained by some degree of washout. There is a concentration gradient of glucose outside the cells, and the supply to the bone may be limited; glycerol is only liberated when there is damage to the cell membrane. A fall in concentration of glycerol may also be part of the washout, due to the high flow rate in the microdialysis catheter. The pyruvate concentration is expected to decrease along with glucose, as this is the substrate for the formation of pyruvate. This might also be part of the explanation for the increase in the lactate/pyruvate ratio over the time in the in-vivo experiment, from a ratio of 30 to 37.

For most substances, the equilibrium across the semipermeable membrane is incomplete. A way of measuring the effectiveness of the microdialysis catheter is to calculate relative recovery (RR). This is the fraction collected in the dialysate divided by the concentration in the interstitial fluid in the tissue that is being investigated. The equation for calculating RR is:


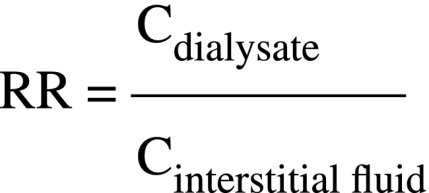


where C_dialysate_ is the concentration in the microdialysis dialysate and C_interstitial fluid_ is the concentration in the extracellular fluid. In practice, RR is calculated from C_dialysate_/C_plasma/serum_ since the plasma concentration is the only measure available.

A relative recovery of 57% is in accordance with the results obtained in our study on human femoral heads (Bøgehøj et al. 2007). The rather low RR compared to other studies (Rooyackers et al. 2004), where almost complete recovery has been reported, is most likely due to the high flow rate of 2 μL/min. This high flow rate was chosen to be sure to get a sufficient amount of sample for the analysis and to compare with human data where the same flow rate was used.

Another interesting finding is that the glycerol was washed out in both parts of the present studies. Compared with the study done on human femoral heads (Bøgehøj et al. 2007), where the glycerol concentration increased over the sample period as the bone became more and more ischemic, we did not find any signs of cell damage in the in-vivo drill hole study. This suggests that the measurements from the in-vivo study were from the bone and not just from a shell of damaged bone around the drill canal.

In summary, we have shown that microdialysis can allow measurement of the concentrations of lactate, pyruvate, glucose, and glycerol in bone reflecting local metabolism, since the measurements indicate that we are not only measuring from a blood clot in the dead space around the catheter in the drill canal. For measurements performed over a long period (of hours), we suggest that there should be an equilibration period (1–2 hours) and a lower flow rate. For sampling over a short time, where an equilibrium period is not possible (during surgery), a recovery rate of 55% can be obtained and washout does not appear to pose any problem. Thus, a reference measurement will be necessary, e.g. in another part of the bone where the blood supply is intact.
